# PRISME: A MATLAB Toolbox For Large Data-Driven Multimodal Power Benchmarking

**DOI:** 10.64898/2026.01.21.700878

**Published:** 2026-01-22

**Authors:** Fabricio Cravo, Alex Fischbach, Hallee Shearer, Matt Rosenblatt, Dustin Scheinost, Stephanie Noble

**Affiliations:** 1 Department of Psychology, Northeastern University, Boston, MA; 2 Department of Bioengineering, Northeastern University, Boston, MA; 3 Center for Cognitive & Brain Health, Northeastern University, Boston, MA; 4 Department of Radiology & Biomedical Imaging, Yale University, New Haven, CT; 5 Department of Biomedical Engineering, Yale University; 6 Interdepartmental Neuroscience Program, Yale University; 7 Child Study Center, Yale School of Medicine; 8 Psychiatric and Neurodevelopmental Genetics Unit, Center for Genomic Medicine, Massachusetts General Hospital, Boston, MA, USA; 9 Center for Precision Psychiatry, Massachusetts General Hospital, Boston, MA, USA

## Abstract

Low statistical power in neuroimaging often undermines research in the field, leading to missed effects, wasted resources, and reduced reproducibility. Performing power analyses during the study design phase is extremely important, but often prohibitively difficult due to a lack of analytical solutions and high computational costs. We present PRISME (Power Resampling Infrastructure for Statistical Method Evaluation), a MATLAB toolbox for neuroimaging power benchmarking. PRISME provides a computational framework for empirical power analysis independent of inference methods, enabling large scale power benchmarking and method comparison. The toolbox supports diverse neuroimaging data types, including both voxel-based activation and functional connectivity analyses, with a non-parametric, flexible algorithm and unified data representations. Furthermore, unlike previous empirical power approaches, PRISME supports multiple test types, such as association and difference tests with behavioral and clinical measures. Finally, PRISME‘s 25× speedup from algorithmic optimizations enables larger-scale power benchmarking, including the first power analysis for the ABCD dataset. Overall, PRISME is the first method- and data-type-agnostic power benchmarking tool for neuroimaging, providing a single solution for power analysis across diverse study designs.

## 1 Introduction

Answering research questions in neuroscience requires reliably detecting statistically significant patterns called true effects in complex brain imaging data despite substantial noise. [FHW^+^94, MTR^+^12, Hay15, VDHP10, PNPH99, SN18, CB24b, CWY17]. Statistical power, the probability of detecting true effects through methods that compare data to randomness (i.e. statistical inference methods), falls far below the recommended standard of 80% in neuroimaging [NMZS22, SN18, CWY17, BIM^+^13, LAH^+^16], with experiments typically achieving only 8–31% average power [BIM^+^13]. This widespread low power leads to several problems for neuroscience research. First, researchers frequently miss key effects that would validate their hypotheses, resulting in inconclusive findings and inefficient usage of time and resources [BIM^+^13, VKS16, Ell22]. Additionally, low statistical power severely hinders scientific reproducibility, which undermines the ability to distinguish genuine findings from false positives. For example, when an effect has merely 10 % power, only one in ten experimental repetitions is expected to detect it reliably [Coh13]. This may partly explain why replication attempts of neuroimaging studies often fail to reproduce previously reported findings [Ell22, Hen20, MHH18].

A known solution to the low power lies in performing proper a-priori power calculations [SN18, Yeu18]. Through proper power estimation during study design, researchers can determine the sample sizes necessary to reliably detect true effects, identify the magnitude of effects their study is powered to detect, and substantially reduce the probability of missing meaningful results [SN18, SRY^+^24]. Studies that incorporate proper power calculations before data collection demonstrate notably improved research quality, with Mumford [Mum12] showing that the process of conducting power analysis frequently leads researchers to refine their study design and improve experimental tasks. Despite these benefits, the number of studies performing power calculations is concerning. Szucs and Ioannidis [SI20] argued for the importance of power calculation for appropriate allocation of research funding and found that only 3% of studies reported a-priori power calculations in 2017–2018.

Despite the importance of power, the approaches for its estimation in neuroimaging are lacking and do not cover the needs of the field. Some of these are parametric approaches [Mum12, DG02, HPHL07, MN08, DDM^+^16, KMLS09], many of which remain methodological frameworks without implemented software tools [DG02, KMLS09] or have tools that are no longer maintained [Mum12, MN08]. Parametric methods require prior assumptions about the shape of the distribution and the nature of the data to find analytical solutions for calculating power, with different assumptions required for a power calculation for each statistical inference method [Coh13]. If experimental data do not follow these assumptions, the power calculation results will be inaccurate. Furthermore, some popular statistical inference methods in neuroscience, such as the Threshold-Free Cluster Enhancement (TFCE) [SN09] and cluster-size inference [WF95], do not have parametric methods available because they currently lack analytical solutions. In addition to these issues, parametric methods require researchers to specify expected effect sizes a priori, increasing the chances of erroneous power estimation. These limitations increase the likelihood of user errors in power estimation and restrict applicability. Non-parametric methods mitigate these issues by estimating power empirically without requiring distributional assumptions, effect size specification, or analytical solutions for each inference method.

Recognizing these advantages, researchers have developed valuable non-parametric methods for estimating power [NSC20, NMZS22]. Noble et al. [NSC20, NMZS22] proposed a non-parametric subsampling repetition algorithm that uses large datasets to simulate experiments with smaller sample sizes and identifies true effects. Despite its methodological strengths, this approach is not productionready for widespread use: it lacks computational efficiency, has no standardized framework for method comparison, and is incompatible with diverse data types, datasets, and test types. Even on a computational cluster, it has high computational demands, requiring on average 64 hours without parallelization for just 500 repetitions per study, make it prohibitively expensive for large-scale analyses. Applied to the ABCD dataset with 40 FC studies across 4 different sample sizes (200, 500, 1000, 2000), this would require approximately 1.1 months of computation even with 16 processor cores under highly optimistic computational assumptions (perfect parallelization and equal fitting cost across test types), qualifying the calculation as impractical. Its implementation is hard-coded to Human Connectome Project (HCP) task-based FC studies, lacking support for other datasets, test types beyond the one sample t-test, and voxel activation-based analysis.

Finally, while the previous non-parametric algorithm has been used to compare methods [NSC20, NMZS22], it uses built-in hard-coded inference methods and it has no capability for users to add their own methods easily for analysis. Therefore, there is no standardized framework available for benchmarking multiple statistical inference procedures. Researchers developing new statistical inference methods have no standardized platform to evaluate their methods against established alternatives under identical conditions and would need to code comparisons by themselves with no guarantee of correctness and standardization. Without such a platform, the field lacks an objective basis for determining which statistical methods offer genuine power improvements in detecting neural effects across different experimental contexts.

We present PRISME, a MATLAB toolbox for efficient empirical power benchmarking in neuroimaging. PRISME addresses the computational and flexibility limitations of previous approaches through several innovations. First, permutation recycling across methods reduces algorithmic complexity from *O*(Methods × Permutations) to *O*(Methods + Permutations), allowing larger scale and faster power analysis. Second, algorithmic optimizations for widely-used methods like cNBS (134× speedup) and TFCE (34× speedup), combined with support for one-sample, two-sample, and correlation analyses, allow for empirical power analysis at a new scale. We demonstrate this with power estimates across 40 ABCD studies, including brain-behavior correlations and group comparisons. Finally, PRISME provides a modular architecture that allows users to add new statistical inference methods easily with a single class script, facilitating the benchmarking of newly developed methods. In the supplementary information, we provide formal mathematical proof of the algorithm’s core concepts relevant for power sub-sampling estimations.

## 2 Results

In this section, we summarize PRISME’s algorithm, computational optimizations, and validation results. First, we describe the subsampling-based power estimation algorithm and the statistical inference methods currently implemented (Section 2.1.2) with the initial GLM fit performed prior (Section 2.1.1). Then, we detail computational optimizations that enabled the large-scale power analysis (Section 2.2). Next, we describe PRISME’s method- and data-agnostic design. Finally, we validate PRISME by replicating previous empirical power estimations for the HCP dataset and extending to 40 ABCD studies and HCP voxel data.

### 2.1 Algorithm

#### 2.1.1 Mass Univariate Regression

PRISME performs mass univariate regression by fitting the same statistical model independently to each brain variable (edge or voxel). It uses the multi-stage General Linear Model (GLM) [WF95, NH02, NS20, SN09, ZFB10], generating one t-statistic per variable for the entire subject group.

Let $Y_{i}\in R^{1\times n}$ be the data matrix containing measurements for variable numbered i (edges or voxels) across n subjects, and $X\in R^{n\times b}$ the design matrix with b model parameters. The design matrix can be a column of ones for one-sample t-tests, a two-column indicator matrix for two-sample t-tests, or continuous measures with an intercept for correlation-based tests. For each variable, we perform the following fit:

(1) $$Y_{i}=X\beta_{i}+\varepsilon_{i}$$

where $\beta_{i}\in R^{b}$ are the model parameters fit for variable numbered i, and $\varepsilon_{i}\in R^{1\times n}$ contains the error terms. From each $\beta_{i}\in R^{b}$, we compute a single t-statistic value. Higher t-statistics indicate stronger evidence against the null hypothesis of no effect. An effect is detected when the null hypothesis has been rejected.

#### 2.1.2 Architecture

**Figure 1:**
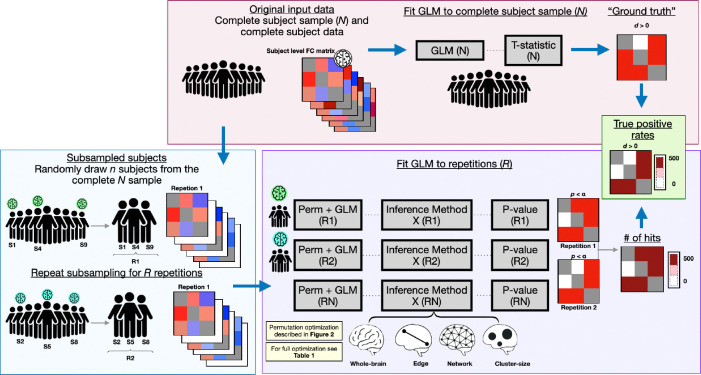
Algorithm workflow for non-parametric power estimation. The workflow comprises two paths for power calculation. **Pink panel**: Complete subject sample path uses all subjects to fit GLM and establish ground truth effect directions based on t-statistic signs. **Blue panel**: Repeated subsampling path randomly draws n subjects from the full dataset across R repetitions. **Purple Panel**: The GLM is fit to both original and permuted data to generate t-statistics, then applies statistical inference methods that produce p-values at different levels (whole-brain, network, edge, cluster-size). Permutations are recycled across all methods for computational efficiency (complexity reduction from O(R×n×M) to O(R×(n+M))). **Green Panel**: Power calculation compares detected effects from each repetition against ground truth, and the statistical power is the proportion of correctly detected effects

The power estimation procedure implemented in PRISME is structured around repeated subsampling to evaluate the ability of statistical inference methods to detect effects known to be present in the full dataset (Figure 1). To do so, it utilizes two distinct computational paths: a repeated-sampling loop that evaluates the methods in multiple subsets of the data, and a single estimation step that defines the ground truth effect map. The results of both paths are then combined to provide error rate estimates.

In the repeated-sampling path, n subjects are randomly sampled without replacement from the larger pool of N available subject-level connectivity matrices. The t-statistics are obtained with mass univariate regression in accordance to Section 2.1.1, which are then used by the statistical inference methods described below. The statistical inference methods, in turn, produce p-values for each repetition. For each repetition, p-values are compared to a significance threshold to identify statistically significant effects (i.e., where the null hypothesis is rejected and effects are detected)

#### 2.1.3 Statistical inference methods

PRISME evaluates statistical power at three inference levels:

- **Variable level**: Power to detect effects in individual analysis units: FC links (edges) between brain regions for FC data, or individual voxels for activation data.
- **Cluster level**: Power to detect effects in spatially contiguous groups of variables through pooled inference (e.g., using Cluster Size or TFCE methods)
- **Network level**: Power to detect aggregate effects within variables in predefined large-scale functional systems (e.g., default mode, salience networks)
- **Whole-brain level**: Power to detect any significant effect across all variables, i.e. if an effect can be detected in any of the studied variables.

The number of p-values that each repetition produces depends on the inference level: one per variable (variable-level and cluster-level methods), one per network (network-level methods), or a single value (whole-brain inference).

Furthermore, several statistical inference methods representing different levels of brain analysis have currently been implemented for use by PRISME. At the individual variable level (single brain connections or voxels), the **Univariate (Parametric)** method calculates p-values by comparing observed t-statistics to the standard t-distribution [WF95, NH02]. **Cluster Size** inference first thresholds the map at a pre-defined statistical threshold, then groups remaining connected variables into clusters and determines significance based on cluster size compared to permutation-generated null clusters [WF95]. **Threshold-Free Cluster Enhancement (TFCE)** combines information from each variable with its neighboring variables and creates spatially-weighted statistics that reflect both local activation strength and spatial extent [SN09]. At the Network level, the **Constrained Network-Based Statistic (cNBS)** pools statistical information from predefined brain networks by averaging t-statistics within each network and comparing these averages against permutation-based null distributions [NS20, ZFB10]. At the whole-brain level, the **Multivariate-cNBS (mv-cNBS)** uses cNBS statistics to form a single vector test statistic; like other multivariate tests, this test reveals a joint effect across brain areas but not at any particular spatial location [NH02]. For methods involving multiple tests, both **False Discovery Rate (FDR)** and **Family-Wise Error Rate (FWER)** corrections are implemented [BH95] according to number of tests at each inference level. FDR controls the expected proportion of false positives among significant results, while FWER controls the probability of making at least one false positive. In total, counting correction methods separately, seven inferential procedures are currently implemented (Parametric-FDR, Parametric-FWER, cNBS-FDR, cNBS-FWER, Cluster Size, TFCE, and mv-cNBS).

#### 2.1.4 Ground truth effects

Separately, ground truth effects are computed using the full sample of N subjects by fitting the group-level GLM and computing t-statistics for all variables, and averaged across networks for network-level inference. With the explicit assumption that ground truth effects define true effects [NMZS22], edges and networks are labeled as true effects in the direction of their t-statistic sign. As noted by Turner et al. [TPMB18], ground truth estimated from finite samples may not precisely capture all effect sizes. However, this provides conservative power estimates: including uncertain small effects in ground truth increases detection difficulty, thereby evaluating the capability of inference methods to detect weak effects. For whole-brain level inference, there are no directional effects. If the ground truth effects indicate that any variable has a non-zero t-statistic, then the positives resulting from inference methods at this level are considered true positives.

#### 2.1.5 Ground truth and Sub-sampling Comparison

At the end of the workflow, detected effects from each repetition are compared to ground truth effects. Statistical power for a given brain area or whole-brain test is defined as the proportion of correctly identified effects across all repetitions. This yields power probability estimates for all inference methods for each variables, networks, or whole-brain according to the method’s inference level.

#### 2.1.6 Power Definitions

In this section, we formally describe the equations used for power calculations.

##### Variable and Network Level Power:

Let R be the number of repetitions. Let x be a variable (edge or voxel) or network, and let $t_{GT}(x)$ denote the t -statistic for variable x computed from the full sample of N subjects For each repetition r∈{1,…,R}, let $D_{r}(x)\in \{-1,\;0,\;+1\}$ indicate whether a negative effect, no effect, or positive effect was detected for variable x.

Power for variable x is defined as:

(2) $$Power(x)=\left\{\begin{array}{ll} \frac{1}{R}\sum_{r=1}^{R}\;1_{\left\{1\right\}}\left(D_{r}(x)\right) & \text{if}\;t_{GT}(x)>0\\ \frac{1}{R}\sum_{r=1}^{R}\;1_{\left\{-1\right\}}\left(D_{r}(x)\right) & \text{if}\;t_{GT}(x)<0\\ \text{Does}\;\text{not}\;\text{apply} & \text{if}\;t_{GT}(x)=0 \end{array}\right.$$

where $1_{\left\{v\right\}}\left(D_{r}(x)\right)$ denotes the indicator function equal to 1 if $D_{r}(x)=v$ and 0 otherwise, with v∈{−1,+1}. That is, power measures the proportion of repetitions where the statistical inference method correctly detected an effect in variable x in the same direction as the ground truth. Variable x is considered to have no effect when $t_{GT}(x)=0$. For analysis of PRISME applied to variables with no true effect, see Section 7.

##### Whole-Brain Level Power:

For whole-brain inference, if ground truth shows any non-zero effect (i.e., $\exists x:t_{GT}(x)\neq 0$), then power is the proportion of repetitions where any effect was detected. Let $B_{r}(x)\in \{0,\;1\}$ indicate whether an effect was detected or not, the power at the whole brain level is:

(3) $${\text{Power}}_{\text{whole-brain}}=\frac{1}{R}\sum_{r=1}^{R}B_{r}\;\text{if}\;\exists x:t_{GT}(x)\neq 0$$

where ∃ is the existence quantifier. Power is undefined if no effects exist in ground truth (null everywhere).

##### Average Power:

Let X be the set of variables whose variables x have a non zero ground truth t-statistic $t_{GT}(x)\neq 0$. The average power is:

(4) $$Average(X)=\frac{1}{\left|X\right|}\sum_{x\in X}Power(x)$$

where |X| is the number of variables in set X.

### 2.2 Computational Optimizations and Performance Analysis

**Figure 2:**
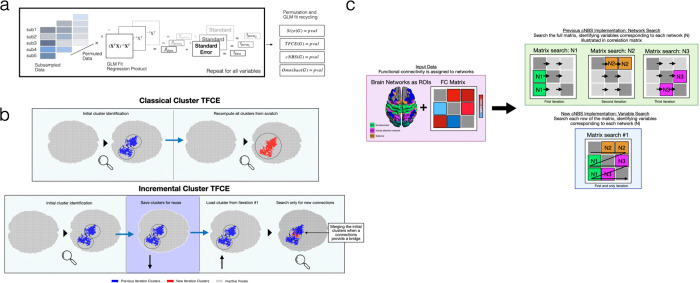
Graphical summary of Computational Optimizations (a) The GLM fit recycling pipeline. The pipeline shows how GLM fits for both data and permutations of the data are generated and recycled for usage in different methods for computational efficiency. (b) Incremental cluster TFCE implementation. Different from traditional TFCE implementations, PRISME computes clusters incrementally for TFCE calculation. (c) cNBS variable level checking. As opposed to the original cNBS implementation that searches for variables within a network for each network, PRISME‘s implementation loops over all variables once and assigns them to their respective network.

PRISME implements several computational optimizations that significantly reduce processing time for non-parametric power estimation. Figure 2 summarizes the computational optimizations and their effects. The key optimizations enabling these performance improvements include:

- **Permutation and GLM fit re-utilization:** Rather than re-fitting the GLM at each permutation for each statistical procedure, PRISME performs a single set of permutation-based GLM fits that are then shared across all inference methods. This reduces computational complexity from *O*(Repetitions × Methods × Permutation) to *O*(Repetitions × (Methods + Permutations))
- **Multiple Correction Methods Applied Simultaneously:** When an inference method supports multiple correction procedures (FWER and FDR), uncorrected p-values are computed once, and both corrections can be applied to the same output. This sub-method architecture avoids running the inference method twice, once per correction type.
- **Batch Processing with Check-pointing:** The repetition loop is segmented into configurable batches that automatically save intermediate results after each batch completes. The batch size allows users to control the amount of repetition-related data loaded into RAM for efficiency, with higher batch sizes requiring more RAM. PRISME can resume calculations from the saved data to provide resilience against system failures and allow computations to be performed across multiple sections.
- **Permutation Recycling Facilitates C++ Integration:** Statistical inference methods can be among the most computationally intensive components of PRISME power calculations, particularly for methods such as TFCE. The class-based architecture enables methods to be prototyped in MATLAB and then replaced with optimized C++ implementations. Since all methods receive identical permutation data from PRISME‘s recycling component, MATLAB and C++ versions must produce identical outputs when given the same inputs. This direct comparability enables confident validation and replacement of performance-critical code
- **Algorithm Optimizations:** PRISME provides faster versions of statistical inference methods. For cNBS, the implementation processes each variable once and assigns it to its network, rather than searching all variables for each network. This reduces the computational complexity from *O*(Networks × Variables) to *O*(Variables). The TFCE algorithm uses an incremental cluster approach that reuses previously computed clusters to compute subsequent threshold clusters. These algorithmic improvements, combined with C++ implementations, provided substantial speedups compared to previous implementations [NS20, SN09] (Table 1).

To show PRISME computational speed improvements, we performed a computational speed benchmark test. We first conducted a power calculation using the HCP dataset for FC with 35778 edges (Shen 268 atlas [STPC13]) for 5 studies across 5 sample sizes (*n* = 20,40,80,120,200). Afterwards, we used the reported average time per study reported by Noble et al. [NMZS22] for an estimation of the total time to conduct the same power calculation under similar computing conditions. The estimated time using Noble et al.’s [NMZS22] approach was 20 days, compared to 20 hours with PRISME resulting in a 25× speed up factor (Table 1). Supplementary Method 6.5 contains more details about the benchmark test and computing conditions.

The computational advantages would be greater for other test types. HCP studies primarily use one-sample t-tests requiring the matrix inversion of a single column design matrix, while ABCD studies include two-sample t-tests and correlation analyses with 2-column design matrices that require more computational time to compute the matrix inversion. As PRISME recycles GLM fits across all inference methods rather than recomputing for each method, the efficiency gains for ABCD datasets would exceed the 25× speedup benchmarked on HCP. However, it is impossible for us to compare this as Noble et al.’s was not capable of performing these tests.

### 2.3 Method Agnostic Approach

**Figure 3:**
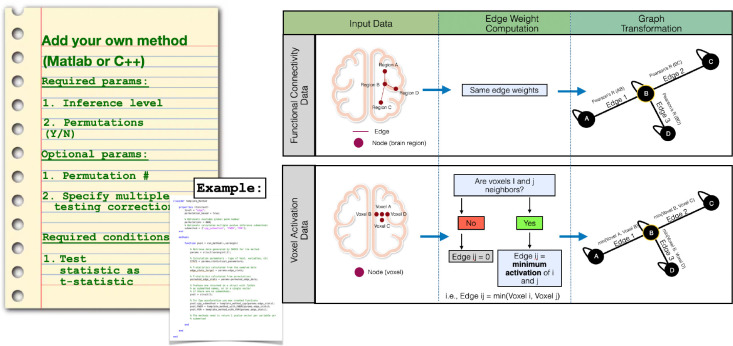
Method- and data-agnostic design of PRISME. *Left*: Method-agnostic interface that allows easy addition of new statistical inference methods. New methods require only specification of inference level (edge/network/whole-brain), permutation requirements, and the core statistical function mapping t-statistics to p-values. *Right*: Data-agnostic method to represent data for methods that require topological information. The figure shows how to process both functional connectivity and voxel activation data through a unified graph transformation. Functional connectivity data map brain regions to graph nodes with correlation strengths as edge weights. Voxel activation data map individual voxels to nodes, with edge weights computed as the minimum activation between spatially adjacent voxels.

The PRISME toolbox provides the first method-agnostic design within inference levels for neuroimaging power analysis (Figure 3). All that is required is that a method generate p-values from t-statistics at one of the supported inference levels. This method-agnosticism arises from two key design principles. First, method-agnosticism is an inherent consequence of the subsampling approach. By repeatedly subsampling a small number of subjects from the full dataset, the algorithm recreates experimental conditions where any statistical inference method can be applied. The statistical power of each method is then evaluated against ground truth effects estimated from the complete dataset. Second, PRISME possesses a modular architecture that facilitates the addition of new methods. Inference methods can be added as new classes that receive t-statistics from upstream code and return p-values.

### 2.4 Data Agnostic Approach

PRISME implements a data-agnostic framework that handles diverse neuroimaging data types through a single pipeline (Figure 3). The data-agnosticism stems from two design principles: flattened array representation and graph-based spatial transformations to abstract statistical inference methods that rely on topological information.

#### 2.4.1 Unified Data Representation

PRISME processes both network and image data using flattened arrays, treating FC edges and voxels as the same kind of input variables (Figure 3). The flattened data are able to be directly used without modification by methods that require no spatial information across data types (Parametric, cNBS, and Omnibus).

#### 2.4.2 Graph-Based Spatial Transformation

For inference methods requiring topological relationships (TFCE and Cluster Size), PRISME employs a graph-based transformation that converts both data types into an identical graph structure. This transformation allows the same algorithms to operate on either data type by mapping different data types to a common data representation.

The graph construction differs by data type and here we introduce the construction:

##### FC data:

Regions of interest become graph nodes, and the FC data between these regions become weighted edges in the graph, with connectivity strengths as edge weights.

##### Voxel activation data:

In traditional voxel-based analysis, TFCE and cluster-size methods first threshold the brain at a statistical cutoff (e.g., t>2.5), removing voxels below this threshold, then identify spatially contiguous groups of above-threshold voxels as clusters. A cluster is a set of voxels where each voxel is spatially adjacent to at least one other voxel in the set.

Our graph representation achieves this by creating a graph where voxels become nodes, spatial adjacencies become edges, edge weights equal the minimum activation between connected voxels, and clusters, equal to the FC representation, are connected subgraphs. As an example, consider a case where two spatially adjacent voxels have activations of 3 and 2. In the graph representation, both those voxels would be connected by an edge with the minimum weight of 2. If the threshold is 2.5, this edge falls below the threshold and is excluded from the graph, resulting in the voxels not being connected anymore and not forming a cluster. This ensures the graph-based cluster detection produces identical results to traditional spatial clustering.

Furthermore, PRISME precomputes voxel neighborhood relationships during initialization, storing them as a sparse adjacency matrix that represents the graph. This one-time preprocessing enables a fast construction of weighted graphs from flattened activation data using the precomputed adjacency structure.

### 2.5 Power Values and Validation

We validated PRISME through two methods. First, we verified that the implementation of statistical inference methods accurately detects known effects using synthetic datasets for each test type (one-sample, two-sample, and association tests). This testing framework confirmed 100% detection accuracy for known synthetic effects and provides method developers with a sanity check for new implementations. Furthermore, this testing framework is implemented as both a standalone script and as a GitHub action.

**Figure 4:**
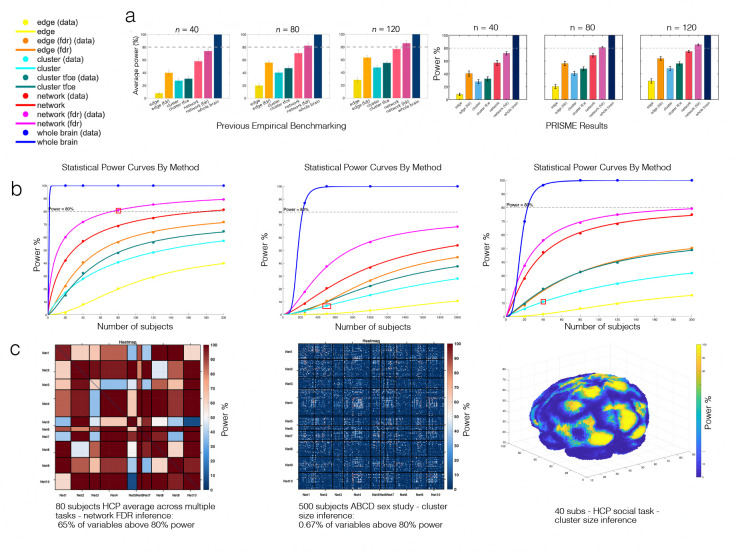
Power analysis results across datasets and methods. (a) Validation of PRISME against previous results from Noble et al., showing average statistical power across different sample sizes (n = 40, 80, 120) for seven inference methods. (b) Statistical power curves across three analysis contexts. Left: HCP task-based studies showing power curves fitted to validation data. Middle: ABCD correlation studies (cognitive-FC relationships). Right: Voxel-based analysis showing lower average power but highlighting spatial heterogeneity in effect detection. Red squares indicate the specific sample sizes and method referenced in panel (c). (c) Detailed spatial patterns of specific power analysis.

Second, we replicated and extended the HCP power analysis from Noble et al. [NMZS22] (Figure 4). We analyzed 5 FC task studies using 7 inference methods (Parametric-FDR, Parametric-FWER, cNBS-FDR, cNBS-FWER, Cluster Size, TFCE, and mv-cNBS) across 4 sample sizes (n=250,n=500,n=1000,n=2000) and compared to their original 3 sample sizes (n=40,n=80,n=120).

By visually comparing the histogram plot provided in Figure 4, one can see that the power results from Noble et al. [NMZS22] closely match the results produced by PRISME. A direct match is not expected due to the stochastic nature of both approaches for power benchmarking.

For voxel-level analyses, we generated power curves across sample sizes for five HCP tasks (n=20, 40, 80, 120, 200; 100 repetitions). This extends Noble et al. [NSC20], who measured detection rates for different effect sizes at n=20, by determining required sample sizes for 80% power and providing voxel-wise power estimates. We additionally evaluate inference methods not included in their analysis: parametric-FDR, parametric-FWER, cNBS-FDR, cNBS-FWER, and mv-cNBS.

Herein a study is considered a valid and available pairwise comparison between two types of data present in a dataset. For example, FC rest data compared to behavior data as correlation, FC rest data versus task data, and FC rest data versus categorical variables.

Having validated the power benchmarking accuracy of PRISME, we demonstrate its computational capability by performing power analyses at a scale previously infeasible: 40 ABCD studies [CCC^+^18, VKC^+^18] (compared to 5 HCP studies in the validation) encompassing both two-sample t-tests (sex differences) and correlation tests (age, cognitive, and behavioral measures). Complete power results for all 40 studies are provided in Supplementary Table 6.2. Figure 4C presents the study with highest observed power: sex differences in resting-state FC, where all methods except cluster size and edge-level parametric FWER exceed 80% power at n=200.

Finally, we provide a mathematical proof that validates PRIME sub sampling method for power estimation for broad application across multiple data types and statistical inference methods in the supplementary information.

## 3 Methods

### 3.1 Implementation and Availability

PRISME is implemented as an open-source MATLAB toolbox (version 2024a or later) distributed under the MIT license via GitHub https://github.com/neuroprismlab/PRISME-Brain-Power-Calculator. The toolbox operates on neuroimaging data in matrix format, with input data structure specifications and data preprocessing detailed in Supplementary Methods. PRISME automatically infers the appropriate statistical test type (one-sample, two-sample, or correlation analyses) based on the input data structure. A usage tutorial is available in the Supplementary Methods. Preprocessed neuroimaging data from the Human Connectome Project and Adolescent Brain Cognitive Development Study in PRISME-ready format can be obtained from the authors upon verification of approved data access from the respective repositories.

### 3.2 Statistical Inference Method Implementation

The method agnostic approach of PRISME is further strengthened by the simplicity of adding new methods for power benchmarking. The modular architecture allows users to add new inference methods simply by adding a new MATLAB class script with the following attributes:

- The inference level (ex: variable, network, etc …)
- A boolean specifying if permutations are required
- The statistical function converting t-statistics to p-values

This feature facilitates the addition and benchmarking of new methods with minimal code. By handling data processing, permutation generation, and ground truth estimation, PRISME allows method developers to focus on statistical innovation while ensuring fair power benchmarking.

## 4 Discussion

PRISME is the first general-purpose power benchmarking tool for neuroimaging. Unlike parametric approaches restricted to specific inference methods or previous empirical approaches limited to single data types, PRISME works across multiple methods, data types, and study designs. The algorithm evaluates any inference method by testing its ability to detect effects in subsampled data against ground truth estimated from the more precise full dataset estimation. New methods can be integrated by creating a single new class. PRISME handles both functional connectivity and voxel activation data through a unified framework, supporting one-sample, two-sample, and correlation analyses. For the first time, researchers have a power benchmarking tool for neuroimaging inference methods and data types within a single framework.

Empirical power estimation usually requires extensive computational time and resources, limiting some practical applications. PRISME addresses this through three key optimizations. First, permutation recycling shares GLM fits across all inference methods rather than recalculating for each, reducing complexity from *O*(Methods×Permutations) to *O*(Methods+Permutations). Second, some methods are implemented in C++ while maintaining MATLAB compatibility. Third, algorithmic improvements to established methods (cNBS, TFCE) provide additional speedups. Together, these achieve a 25× overall speedup compared to previous empirical approaches, reducing times from weeks to days.

We validated PRISME through several analyses. We replicated Noble et al.’s [NMZS22] HCP power analysis, producing power estimates matching previously established results. We used the new computational efficiency to benchmark power on 40 ABCD studies across 4 sample sizes, a tenfold increase in scale compared to previous benchmarking with 5 HCP studies and 3 sample sizes [NMZS22]. Finally, we demonstrated the data-agnostic capability through power analysis of voxel task-activation data. Together, these analyses establish PRISME as a general-purpose solution for empirical power estimation in neuroimaging.

The PRISME toolbox can extend naturally to other neuroimaging modalities that employ similar statistical approaches. For example, electroencephalogram (EEG) studies utilize functional connectivity analysis [CSA^+^23]. Similarly, functional near-infrared spectroscopy (fNIRS) applies GLM frameworks for task activation and functional connectivity estimation [HZZ^+^21] equally to fMRI. The difference is that fNIRS measures cortical activity near the skull surface with fewer variables than fMRI. These modalities may require only minor data format conversions, or potentially could work with PRISME directly without modification. We plan to formalize support for these modalities in future releases.

The ground truth estimation has important limitations. Statistical power is known to increase with the magnitude of the underlying true effect and the sample size [Coh88]. With PRISME, we assume the ground truth estimation is the true effect for the entire population. The ground truth effects are estimations from a true effect sampling distribution, whose estimation accuracy increases with the number of subjects in the available dataset [CB24a]. This can lead to errors when the ground truth is not a precise enough estimation of the true underlying effect. These estimation errors directly affect measured power because we measure detection probability relative to the ground truth estimate.

For ground truth effects with incorrectly estimated signs, recent analyses of large neuroimaging datasets reveal that confidence intervals for many effects overlap zero, indicating uncertainty in the true effect sign [SRY^+^24]. This occurs more frequently for low-magnitude effects. PRISME partially mitigates this by independently testing for both positive and negative effects. When ground truth incorrectly assigns an effect’s sign, the inference method is more likely to fail to detect the effect across repetitions due to the low magnitude. This results in conservative power estimates, ensuring that actual observed power is more likely to exceed our estimates than fall below them.

PRISME addresses a critical gap: the lack of accessible and versatile tools for empirical power analysis by providing the first method- and data-agnostic power benchmarking tool for neuroimaging. With PRISME, researchers can perform power calculations for sample size planning, and the framework enables standardized benchmarking of statistical inference methods under equivalent conditions. As neuroimaging continues to grapple with reproducibility challenges, PRISME facilitates proper study planning and methodological evaluation to improve research quality. We anticipate that wider adoption of empirical power analysis will contribute to more reliable findings, more efficient resource allocation, and more reproducible neuroscience.

## Supplementary Material

Supplement 1

## Figures and Tables

**Table 1:** Performance gains from algorithmic optimizations

Computational Improvement	Code Component Speedup Factor

*Overall Improvement*	
GLM Fit Recycle (5 permutation methods, positive and negative effect direction)	10×

*Method-Specific Computational Improvements*	
Parametric - using same uncorrected p values for FWER + FDR	~2×
Threshold-Free Cluster Enhancement (TFCE) C++ implementation and incremental cluster computation	34×
Cluster Size C++ implementation	4×
cNBS C++ implementation and variable level check and network assignment	134×

**Overall Performance Gain**	
Full HCP dataset (25 studies, 25 cores)	~25×
